# Complete sequence determination and analysis of mitochondrial genome in *Oreonectes daqikongensis*

**DOI:** 10.1080/23802359.2016.1202739

**Published:** 2016-09-05

**Authors:** Huaiqing Deng, Huamei Wen, Ning Xiao, Jiang Zhou

**Affiliations:** a School of Life Sciences, Guizhou Normal University, Guiyang, Guizhou, China;; b Guiyang Nursing Vocational College, Guiyang, Guizhou, China

**Keywords:** Mitochondrion, *Oreonectes daqikongensis*, phylogenetic tree

## Abstract

*Oreonectes daqikongensi* revealed that the complete length of its mitochondrial genome was 16,578 bp, composed of A (30.7%), T (25.6%), G (16.2%), C (27.5%) and A + T (56.3%). Its genetic constitution and arrangement were consistent with those of other Osteichthyes, including 13 protein-coding genes, 22 tRNA genes, 2 rRNA genes, and 1 control area (D-loop). All genes were encoded by the H-strand, except for 1 protein-coding gene (ND6) and 8 tRNA genes (tRNA-Gln, tRNA-Ala, tRNA-Cys, tRNA-Asn, tRNA-Tyr, tRNA-Ser, tRNA-Glu and tRNA-Pro) are encoded by the L-strand. The mitochondrial genes were arranged very closely. There were four gene overlapping regions, with an overall length of 21 bp and a base covering number range of 1–14 bp, and 23 intergenic regions, with an overall length of 147 bp and an intergenic length range of 1–19 bp. There were 10 gene pairs that were neither overlapping nor intergenic.

## Introduction

*Oreonectes* belongs to the Subfamily Nemacheilinae of the Family Balitoridae, which are found only in southwest China and northern Vietnam (Zhu 1989; Du et al. 2008). Balitoridae and their phylogenetic analyses are very important for studying the environmental adaptability of freshwater fishes (Doadrio & Perdices 2005; Perdices et al. 2012). In this study, complete sequences of mitochondrial DNA of *Oreonectes daqikongensis* were tested. The specimen was collected from a subterranean river of the Daqikong area (25°17′05.1″N, 107°44′54.3″E), Libo county of China in January 2011. It was stored in the animal specimen room of the School of Life Sciences, Guizhou Normal University, Guiyang, China.

## Method

Total DNA was extracted from the fish muscle tissues and 26 pairs of primers were used to amplify genomic DNA. Then, complete mitochondrial genome was submitted into GenBank (Accession no. KU987436)

Complete mitochondrial DNA sequences of 7 genus 31 species of Nemacheilinae were obtained from GenBank, from which Cytb protein-coding genes were extracted. The phylogenetic tree was established using the neighbor-joining (NJ) method, while reliability was tested using the Kimma2-Pamameter method (Kimura 1980) in MEGA 6.0.

## Results and discussion

The overall length of the mitochondrial genome of *O. daqikongensis* was 16,578 bp, including 22 tRNA genes, 2 rRNA genes, 13 protein-coding genes and 1 control area (D-loop). All genes were encoded by the H-strand, except for 1 protein-coding gene (ND6) and 8 tRNA genes (tRNA-Gln, tRNA-Ala, tRNA-Cys, tRNA-Asn, tRNA-Tyr, tRNA-Ser, tRNA-Glu and tRNA-Pro), which were encoded by the L-strand. There were four gene overlapping regions, with an overall length of 21 bp (tRNA-Ile and tRNA-Gln, ATP8 and ATP6, ND4L and ND4, tRNA-Pho and D-loop), with a base covering number range of 1–14 bp, and there were 10 gene pairs that were neither overlapping nor intergenic, while there were 23 intergenic regions, with an overall length of 147 bp and an intergenic length range of 1–19 bp. The complete nucleotide sequence was composed of A (30.7%), T (25.6%), G (16.2%), C (27.5%) and A + T (56.3%), and had a certain degree of AT (66.2%) bias in the D-loop region.

**Figure 1.**
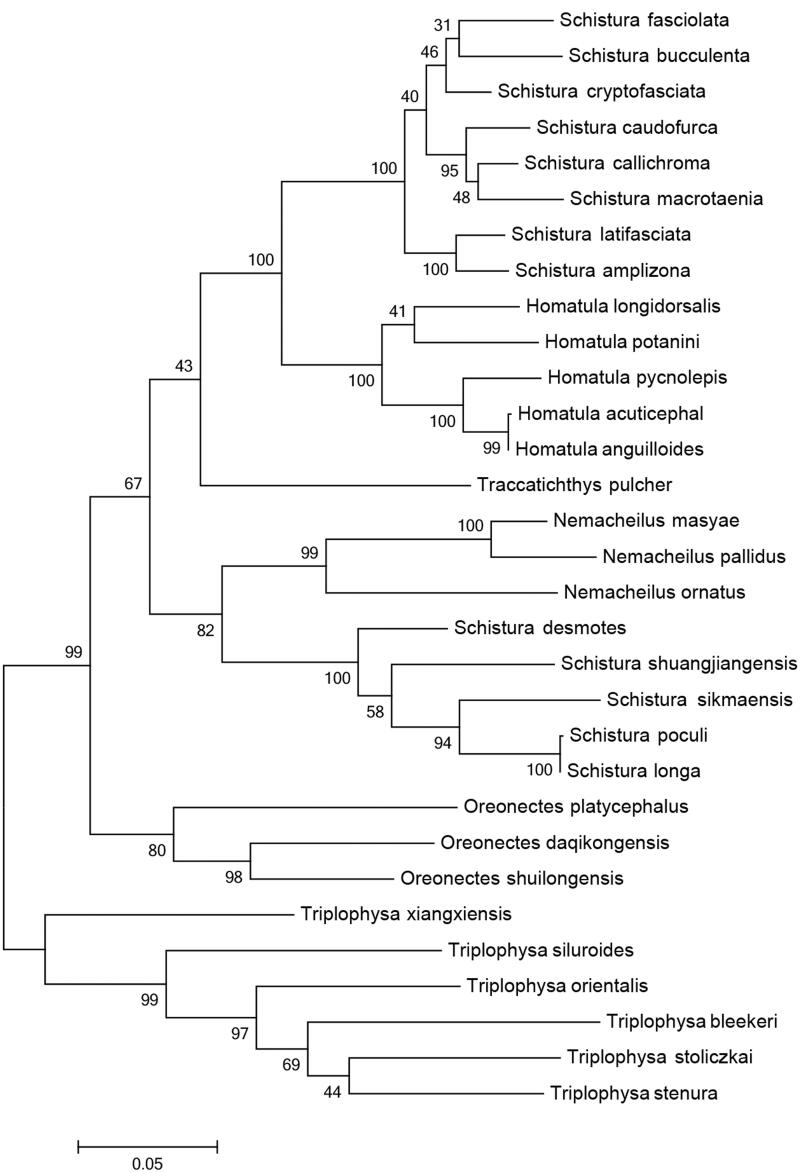
NJ tree established using CytB gene sequence. Genbank accession number for the published sequences are *Oreonectes shuilongensis* (KF640641), *Oreonectes platycephalus* (DQ105197), *Oreonectes daqikongensis* (KU987436), *Schistura fasciolata* (HM010565), *Schistura shuangjiangensis* (JN837651), *Schistura desmotes* (GQ174368), *Schistura callichroma* (JN837652), *Schistura latifasciata* (JN837653), *Schistura bucculenta* (JN837654), *Schistura macrotaenia* (JN837655), *Schistura amplizona* (JN837656), *Schistura cryptofasciata* (JF340401), *Schistura sikmaiensis* (JF340405), *Schistura poculi* (JF340407), *Schistura longa* (JF340408), *Homatula pycnolepis* (KF041000), *Homatula acuticephala* (HM010527), *Homatula longidorsalis* (HM010550), *Homatula potanini* (JF340388), *Homatula anguillioides* (HM010582), *Traccatichthys pulcher* (JF340402), *Triplophysa xiangxiensis* (JN696407), *Triplophysa stoliczkae* (DQ105249), *Triplophysa siluroides* (EF212443), *Triplophysa bleekeri* (FJ406605), *Triplophysa stenura* (JN837657), *Triplophysa orientalis* (DQ105251), *Nemacheilus maysae* (GQ174377), *Nemacheilus ornatus* (GQ174363), *Nemacheilus pallidus* (GQ174370).

The NJ phylogenetic tree established using Cytb gene sequence at the genus level is shown in Figure 1. *O. daqikongensis* was closer genetically to *O. shuilongensis* with a high bootstrap value (BP = 99), than clustered with *O. platycephalus* (BP = 73) supporting the inclusion of *O. daqikongensis* in *Oreonectes*.

Gene sequences in different segments of mitochondrial DNA may have different evolutionary rates (Guo et al. 2004). The ND and Cytb genes are more reliable to establish phylogenetic relationships for class group with further kinship (Zardoya & Meyer 1996). In this study, we used Cytb genes though NJ method to build phylogenetic tree. The results revealed that the fish of Nemacheilinae were divided into two branches. One branch is *Oreonectes, Schistura, Homatula, Paracobitis and Nemacheilus,* which is distributed in the low altitude area. Another branch is *Triplophysa* fishes distributed in the high elevation area. This may be due to the uplift of Qinghai-Tibet plateau which makes the geographical isolation and form different species.
